# Epidemiology and anatomical distribution of stress fractures in children and adolescents: an 8-year retrospective study

**DOI:** 10.1186/s13018-026-06768-6

**Published:** 2026-03-01

**Authors:** Xinjian Pei, Jinchao Cao, Yunshan Su, Junzhong Luo, Jiuhui Han

**Affiliations:** Department of Pediatric Orthopedics, The Third Hospital of Hebei Medical University, No. 139 Ziqiang Road, Shijiazhuang, 050051 Hebei China

**Keywords:** Stress fracture, Children, Adolescents, Epidemiology, Tibia, Consultation-based rate, Anatomical distribution.

## Abstract

**Objective:**

This study aimed to investigate the epidemiological characteristics and anatomical distribution of stress fractures (SFs) in a pediatric and adolescent population over an eight-year period.

**Methods:**

We conducted a retrospective cohort study at a tertiary care orthopedic center. We reviewed medical records and imaging data for patients aged 6–18 years who were diagnosed with SFs between January 2017 and December 2024. Patients were assigned to age groups of 6–11, 12–15, and 16–18 years. We collected demographic data, fracture location, and annual consultation-based rates. Statistical analyses were performed using SPSS 26.0 and R software for trend analysis.

**Results:**

A total of 726 patients (403 males, 323 females; median age 14 years, IQR 12–15) were included. The overall consultation-based rate was 23.20 per 100,000 unique pediatric orthopedic patients. Cases were concentrated in the 12–15 years age group, which accounted for 78.24%. The tibia was the most commonly involved bone (83.06%), and the proximal tibia was the single most frequent site (48.62%). Annual rates rose from 8.09 per 100,000 in 2017 to a peak of 38.98 in 2022, then declined to 23.98 in 2024(Poisson regression for linear trend, *P* < 0.001). Male patients had a higher proportion of proximal tibia fractures, whereas female patients had more mid- to distal-tibia fractures (*P* < 0.01).

**Conclusions:**

SFs predominantly affected adolescent males, and the proximal tibia was the most vulnerable site. The observed rise-and-fall trend temporally coincided with shifts in educational and physical activity policies in China, although causal inference was limited by the ecological study design. These descriptive findings emphasized the need for prospective studies that incorporate exposure and risk-factor assessment to inform targeted prevention strategies.

**Supplementary Information:**

The online version contains supplementary material available at 10.1186/s13018-026-06768-6.

## Introduction

Stress fractures (SFs) are a common overuse injury, accounting for up to 20% of sports-related injuries [1]. They are classified as fatigue fractures, which occur in normal bone exposed to abnormal repetitive stress, or as insufficiency fractures, which occur in weakened bone [2, 3]. Fatigue fractures are especially common in young, active individuals; cyclic, sub-threshold loading disrupts the balance between bone formation and resorption, resulting in microstructural failure [3, 4].

Although extensive research has examined SFs in adult athletes and military personnel [5, 6], epidemiological data on the general pediatric and adolescent population remain relatively scarce. This gap is clinically significant because the pediatric skeleton is undergoing rapid growth and development and is therefore more susceptible to injury from repetitive stress. Moreover, SFs in this age group often have an insidious onset, which leads to delayed diagnosis, prolonged healing, and an increased risk of progression to complete fracture or nonunion. These outcomes can substantially disrupt physical training, academic performance, and psychological well-being [7].

In China, as the education system places greater emphasis on physical fitness, understanding the burden and patterns of SFs in school-aged children has become crucial for guiding prevention strategies. This study performed a comprehensive epidemiological analysis of SFs in patients aged 6–18 years treated at a major orthopedic center over eight years (2017–2024). We examined temporal trends, demographic characteristics, and fracture distribution descriptively, and we discussed potential contextual influencing factors.

## Materials and methods

### Study design and setting

This study was a retrospective single-center cohort study conducted at the Third Hospital of Hebei Medical University, a leading tertiary orthopedic specialty hospital in northern China. The protocol was approved by the Institutional Ethics Committee (Approval No: 2023-021-1). Because the study was retrospective, the requirement for informed consent was waived.

### Participants

Patients were identified by searching the hospital’s Picture Archiving and Communication System (PACS) for all emergency, outpatient, and inpatient records between January 1, 2017, and December 31, 2024. The search used terms including “stress fracture,” “fatigue fracture,” “stress injury,” “periosteal reaction,” and related provisional diagnoses.

### Inclusion and exclusion criteria

Inclusion criteria were: [1] age between 6 and 18 years at diagnosis; and [2] a confirmed diagnosis of SF based on imaging (X-ray, CT, or MRI) and clinical assessment. Exclusion criteria were: [1] age < 6 or > 18 years; [2] imaging evidence of osteoporosis, an underlying bone metabolic disease, or a pathological fracture; and [3] atypical clinical or radiological findings that preclude a definitive diagnosis.

### Data collection and diagnostic confirmation

Two experienced attending orthopedic physicians independently reviewed the medical records and corresponding imaging studies (X-ray, CT, and/or MRI) for all identified cases. The diagnosis of SF was based on established imaging criteria: MRI findings of bone marrow edema on fluid-sensitive sequences, with or without a visible fracture line, were considered diagnostic; CT or radiographic findings of periosteal reaction, cortical lucency, or a visible fracture line were also accepted [8, 9]. “Proximal tibia” was defined as the proximal third of the tibial length, excluding isolated findings consistent with tibial tubercle apophysitis (e.g., Osgood–Schlatter disease) that did not show a clear stress-injury pattern on MRI. “Mid–distal tibia” referred to the middle and distal thirds of the tibial shaft. When the two reviewers disagreed, a senior orthopedic consultant made the final decision. The two reviewers initially disagreed on 38 cases (5.2%), and the senior consultant resolved these disagreements. Inter-rater reliability was excellent (Cohen’s kappa = 0.87). We extracted the following data: age, sex, date of diagnosis, and anatomical location of the SF (laterality and specific bone/segment). The hospital statistics department provided the annual total number of unique patients aged 6–18 years with orthopedic consultations (outpatient and emergency visits), which we used to calculate consultation-based rates. Rates are expressed as cases per 100,000 unique pediatric orthopedic patients per year.

### Statistical analysis

Statistical analyses were performed using IBM SPSS Statistics for Windows, Version 26.0 and R software (version 4.3.0). Continuous data (age) were reported as median and interquartile range (IQR) due to a skewed distribution. Categorical variables are reported as counts and percentages (n, %). Consultation-based rates were calculated as the number of SF cases per 100,000 unique pediatric orthopedic patients within the same age range per year. Annual rates are presented with 95% confidence intervals (CIs) calculated using the Poisson distribution. Temporal trends were evaluated by Poisson regression with calendar year entered as a continuous linear predictor to test for a monotonic increase or decrease over time; the model included the log of the annual unique patient count as an offset. Group comparisons, for example fracture site distribution between sexes, were conducted using the Pearson chi-square test. Odds ratios (ORs) with 95% CIs were calculated for key comparisons. A two-tailed P value < 0.05 was considered statistically significant.

## Results

### Baseline characteristics and consultation-based rates

Between 2017 and 2024, there were 3,129,120 unique pediatric orthopedic patients aged 6–18 years. Of these, 726 patients met the inclusion criteria, yielding an overall consultation-based rate of 23.20 per 100,000 patients. The cohort included 403 males (55.51%) and 323 females (44.49%), with a male-to-female ratio of 1.25:1. The median age was 14 years (IQR 12–15). The age distribution was skewed: 568 patients (78.24%) were 12–15 years old, 128 patients (17.63%) were 16–18 years old, and 30 patients (4.13%) were 6–11 years old. Age- and sex-specific denominators were available and used for subgroup rate calculations (Table 1).

**Table 1:** Demographic and clinical characteristics of children and adolescents with stress fractures (2017–2024)

Characteristic	*n* (%)	Consultation-Based Rate (per 100,000 patients)
Total	726(100)	23.20
Age (years), Median (IQR)	14 (12–15)	-
6–11	30(4.13)	0.96
12–15	568(78.24)	18.15
16–18	128(17.63)	4.09
*Sex*		
Male	403(55.51)	12.88
Female	323(44.49)	10.32
*Fracture Site*		
Tibia (Total)	603(83.06)	19.27
Proximal Tibia	353(48.62)	11.28
Mid-Distal Tibia	250(34.44)	7.99
Distal Femur	77(10.61)	2.46
Metatarsals	26(3.58)	0.83
Fibula	11(1.52)	0.35
Tibia/Fibula	9(1.24)	0.29

Age- and sex-specific denominators were provided by the hospital statistics department. The “Tibia/Fibula” category refers to concurrent shaft fractures of both bones

### Fracture site distribution

The tibia was the predominant site of injury, accounting for 603 cases (83.06%). Within tibial fractures, the proximal third was the most frequent location (353 cases, 48.62% of the total), followed by the mid-distal shaft (250 cases, 34.44%). Other sites included the distal femur (77 cases, 10.61%), metatarsals (26 cases, 3.58%; second metatarsal, *n* = 12; third metatarsal, *n* = 8), isolated fibula fractures (11 cases, 1.52%; predominantly distal third, *n* = 9), and combined tibia–fibula shaft fractures (9 cases, 1.24%). No hip, femoral neck, or pelvic fractures were recorded A significant sex-based difference in the precise location of tibial fractures was observed (*P* < 0.01). Although overall tibial involvement was similar between males (82.88%) and females (83.28%), males had a higher proportion of proximal tibia fractures (59.80% vs. 34.67%; OR = 2.81, 95% CI: 2.09–3.79), while females had more mid-distal tibia fractures (48.61% vs. 23.08%; OR = 3.16, 95% CI: 2.32–4.32) (Table 2).

### Diagnostic imaging modality

Among the 726 confirmed cases, diagnoses were established primarily by MRI in 598 cases (82.4%), by CT in 88 cases (12.1%), and by radiography alone in 40 cases (5.5%). The proportion diagnosed by MRI rose from 75.0% in 2017 to 88.7% in 2022 and then stabilized at about 85% in 2023–2024.

**Table 2:** Comparison of fracture site distribution between male and female patients

Sex	Tibia, *n* (%)	Proximal Tibia, *n* (%)	Mid-Distal Tibia, *n* (%)	Other Sites, *n* (%)
Male (*n* = 403)	334(82.88)	241(59.80)	93(23.08)	69(17.12)
Female (*n* = 323)	269(83.28)	112(34.67)	157(48.61)	54(16.72)
χ²; P-value	0.022; 0.882	46.2; <0.001	54.4; <0.001	0.016; 0.900
OR (95% CI)	0.97 (0.66–1.43)	2.81 (2.09–3.79)	3.16 (2.32–4.32)	1.02 (0.69–1.52)

### Temporal trends in consultation-based rates

**Fig. 1:**
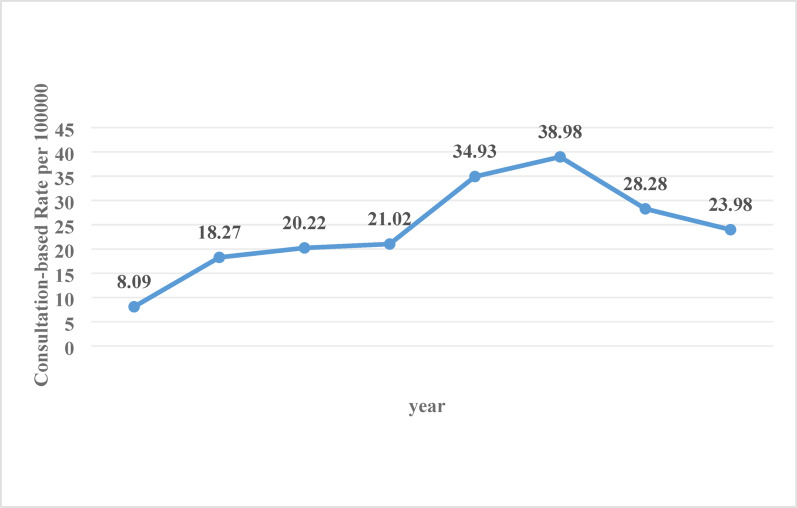
Annual consultation-based rate of stress fractures (per 100,000 unique pediatric orthopedic patients) in children and adolescents (2017–2024)

The annual consultation-based rate exhibited a nonstationary pattern over the eight-year study period (Fig. 1, Supplementary Table S1). The rate increased steadily from 8.09 per 100,000 (95% CI: 6.2–10.5) in 2017 to a peak of 38.98 (95% CI: 32.9–46.0) in 2022. It then declined appreciably in the final two years, falling to 28.28 (95% CI: 23.2–34.3) in 2023 and 23.98 (95% CI: 18.5–30.8) in 2024. Poisson regression confirmed a significant temporal trend (*P* < 0.001). The incidence rate ratio (IRR) per year was 1.21 (95% CI: 1.17–1.26), corresponding to an average annual increase of 21% under a linear model. Visual inspection of the annual rates (Fig. 1), however, indicated a rise-and-fall pattern that the simple linear model did not fully capture.

## Discussion

This eight-year retrospective study presented a detailed epidemiological profile of SFs in a large cohort of Chinese children and adolescents. Our key descriptive findings indicated that SFs predominantly affected adolescents aged 12–15 years, were more common in males, most frequently involved the proximal tibia, and exhibited a non-stationary temporal pattern characterized by an initial increase followed by a later decrease in consultation-based rates between 2017 and 2024.

The predominance of adolescents, particularly those aged 12–15 years (largely corresponding to middle school students in China), aligns with a period of rapid skeletal growth and increased participation in organized sports and mandatory physical training. This pattern contrasts with observations from some Western countries, where SF incidence often peaks in high school or collegiate athletes [10], and highlights the potential influence of region-specific educational and athletic systems on injury patterns.

The male predominance (1.25:1) observed in our study contrasts with reports among adult female athletes and military recruits, where higher SF rates are often reported and commonly attributed to the female athlete triad or Relative Energy Deficiency in Sport (RED-S) [11, 12]. While RED-S is a known risk factor, our study lacked hormonal, nutritional, and energy availability data for assessment. In our population-based sample, behavioral factors may contribute to the sex disparity; boys’ greater participation in high-impact sports, higher training volumes, or different loading patterns may increase their risk [13, 14]. The marked difference in tibial fracture location by sex is noteworthy. The higher proportion of proximal tibia fractures in males may reflect greater forces generated during jumping and high-velocity activities common in sports favored by boys [15]. By contrast, the higher rate of mid-distal tibial fractures in females may stem from different biomechanical patterns or sport preferences, a hypothesis that warrants further biomechanical investigation.

The temporal trend in consultation-based rates is notable. Rates rose steadily from 2017 to 2022, coinciding with increased emphasis on physical fitness testing in the Chinese education system and possibly prompting more intensive student training. The decline in rates after 2022 requires contextual interpretation and must be treated with caution. Although this decline is temporally coincident with national policy shifts (e.g., the ‘Double Reduction’ policy) and post-pandemic societal adjustments [16, 17], our study lacks direct measures of population-level physical activity, training loads, or policy implementation intensity. Alternative explanations deserve careful consideration, including changes in healthcare-seeking behavior, referral patterns to our tertiary center, increased diagnostic sensitivity (particularly with MRI), or shifts in the hospital’s catchment-area demographics over time. Thus, any attribution to specific policies remains speculative and would require integration of external, independent data on relevant exposures for validation.

The predominant involvement of the tibia, particularly its proximal segment, aligns with biomechanical expectations. The tibia is the primary weight-bearing bone of the lower leg and absorbs substantial repetitive stress during running and jumping [18]. The stress concentration in the proximal tibia in adolescents may relate to growth-associated changes in bone density and structure and to forces from muscle attachments [19].

### Limitations

This study had several limitations. Its retrospective design introduced potential biases in data collection and precluded causal inference. The single-center setting may limit generalizability and reflect specific referral patterns. Important exposure and risk-factor data, including detailed activity histories, nutritional status, bone mineral density, and hormonal profiles, were not systematically available. Calculating rates using hospital consultations rather than a defined community population is another constraint; our metric is best interpreted as a hospital workload indicator sensitive to changes in healthcare access and referral practices. Data on school grade and laterality were incomplete and thus not analyzed. Third, our case identification relied on PACS keyword searches. This method may have missed cases coded with alternative terms (e.g., “stress reaction,” “bone marrow edema,” “periostitis”) or cases with atypical symptoms that did not prompt immediate imaging. Although we used broad search terms that included related phrases such as “periosteal reaction,” changes in diagnostic coding or in radiologists’ reporting language over the eight-year period could have introduced time-varying ascertainment bias. The direction of this potential bias is uncertain and may have affected the validity of the observed temporal trend. In addition, the increasing use of MRI—a more sensitive modality for early stress injury detection—over the study period may have contributed to the rise in detected cases, particularly in the early years. This diagnostic surveillance bias cannot be fully discounted when interpreting the temporal trend.

## Conclusions

Despite these limitations, this large-scale study delineated the epidemiological and anatomical distribution of SFs in the Chinese pediatric orthopedic population. SFs were a common clinical presentation, primarily affecting adolescent males and localizing to the proximal tibia. The observed trend in consultation-based rates appeared sensitive to broader socio-educational contexts, although mechanistic explanations remain speculative. These descriptive results called for future prospective, multi-center studies that incorporated detailed exposure and risk factor assessment (e.g., training load, sport type, nutrition, RED-S screening) to confirm associations and to develop effective, evidence-based prevention protocols. Preventive strategies should emphasize educating coaches and adolescents on gradual training progression and should consider sex-specific biomechanical guidance in sports programs [20].

## Supplementary Information

Below is the link to the electronic supplementary material.

Supplementary Material 1

## Data Availability

The datasets generated and/or analyzed during this study are not publicly available because of patient privacy regulations, but they are available from the corresponding author upon reasonable request.
