# Supplementary material for: Epidemiology and anatomical distribution of stress fractures in children and adolescents: an 8-year retrospective study
Source: J Orthop Surg Res. 2026 Mar 1;21:241. doi: 10.1186/s13018-026-06768-6 (PMC13059572; doi:10.1186/s13018-026-06768-6)
Supplement: Supplementary file 1 — Supplementary Material 1 [file 13018_2026_6768_MOESM1_ESM.docx]

****Supplementary Table S1. Annual consultation-based rates of stress fractures per 100,000 unique pediatric orthopedic patients (aged 6–18 years) with 95% Confidence Intervals (CIs), 2017–2024.****

| **Year** | **Number of SF Cases** | **Number of Unique Pediatric Orthopedic Patients*** | **Rate per 100,000 (95% CI)** |
| --- | --- | --- | --- |
| 2017 | 42 | 519,200 | 8.09 (6.2 – 10.5) |
| 2018 | 68 | 372,195 | 18.27 (10.4 – 16.8) |
| 2019 | 95 | 469,832 | 20.22 (15.5 – 23.3) |
| 2020 | 88 | 418,649 | 21.02 (18.6 – 28.5) |
| 2021 | 124 | 354,996 | 34.93 (28.3 – 40.5) |
| 2022 | 142 | 364,320 | 38.98 (32.9 – 46.0) |
| 2023 | 105 | 371,280 | 28.28 (23.2 – 34.3) |
| 2024 | 62 | 258,648 | 23.98 (18.5 – 30.8) |
| ****Total**** | ****726**** | ****3,129,120**** | ****23.20 (22.8 – 26.4)**** |
